# Efferocytosis and Outside-In Signaling by Cardiac Phagocytes. Links to Repair, Cellular Programming, and Intercellular Crosstalk in Heart

**DOI:** 10.3389/fimmu.2017.01428

**Published:** 2017-11-01

**Authors:** Matthew DeBerge, Shuang Zhang, Kristofor Glinton, Luba Grigoryeva, Islam Hussein, Esther Vorovich, Karen Ho, Xunrong Luo, Edward B. Thorp

**Affiliations:** ^1^Department of Pathology, Feinberg Cardiovascular Research Institute, Feinberg School of Medicine, Northwestern University, Chicago, IL, United States

**Keywords:** efferocytosis, heart, phagocytosis, macrophage, cardiomyocyte

## Abstract

Phagocytic sensing and engulfment of dying cells and extracellular bodies initiate an intracellular signaling cascade within the phagocyte that can polarize cellular function and promote communication with neighboring non-phagocytes. Accumulating evidence links phagocytic signaling in the heart to cardiac development, adult myocardial homeostasis, and the resolution of cardiac inflammation of infectious, ischemic, and aging-associated etiology. Phagocytic clearance in the heart may be carried out by professional phagocytes, such as macrophages, and non-professional cells, including myofibrolasts and potentially epithelial cells. During cardiac development, phagocytosis initiates growth cues for early cardiac morphogenesis. In diseases of aging, including myocardial infarction, heightened levels of cell death require efficient phagocytic debridement to salvage further loss of terminally differentiated adult cardiomyocytes. Additional risk factors, including insulin resistance and other systemic risk factors, contribute to inefficient phagocytosis, altered phagocytic signaling, and delayed cardiac inflammation resolution. Under such conditions, inflammatory presentation of myocardial antigen may lead to autoimmunity and even possible rejection of transplanted heart allografts. Increased understanding of these basic mechanisms offers therapeutic opportunities.

## Introduction

Each day billions of cells per person must be cleared during homeostatic cellular turnover (1). Inefficiencies of phagocytic clearance lead to exposure of self-antigen, which is a precursor to autoimmune reactivity (2). In contrast to unicellular organisms that utilize phagocytosis primarily to ingest nutrients, herein we focus on metazoans, and specifically heart tissue, in which the process of phagocytosis by professional phagocytes, particularly macrophages, has evolved additional organ-specific significance, the latter a topic of significant interest in understanding how the local environment shapes cellular identity and tissue homeostasis (3). Prior to just 2014, traditional views held that cardiac phagocytes arose from blood monocytes. However, discoveries on the contributions of cardiac macrophages that have taken up residence prenatally has evolved this view (4) and elevated our appreciation of the diversity of cardiac phagocyte subsets. Herein, this review will discuss our own evolving understanding of phagocyte function in the heart, with particular attention paid to phagocytic signaling, a core phagocyte function with consequences during cardiac development, homeostasis, and disease.

## Phagocytosis in the Developing Heart

The inextricably linked pathways of programed cell death and cellular removal, in turn contribute to tissue remodeling that is integral to embryonic and postnatal organ development. During cardiac development, human fetal cardiocytes appear to ingest other apoptotic cardiocytes, and failure to do so is a hallmark of fetal congenital heart block (CHB) and associated maternal antibodies to ribonucleoproteins (5). For example, binding of antiribonucleoprotein antibodies to apoptotic cardiocytes modifies the distribution of urokinase plasminogen activator receptors, which serves as an antiphagocytic “*don’t eat me*” signal and prevents phagocytosis of apoptotic cardiocytes by neighboring viable cardiocytes (6). Accumulation of these opsonized apoptotic cardiocytes triggers proinflammatory cytokine secretion by macrophages, leading to the fibrosis characteristic of CHB (7, 8). In the case of myeloid phagocytes, and in contrast to the roles of cardiac macrophages in the adult heart, far less is understood on the function of phagocytes during embryonic, fetal, and neonatal stages. Loss of macrophage differentiation or function in the developing heart of *Xenopus* embryos arrests heart formation with targeted depletion of *spib*, a transcription factor essential for primitive macrophage differentiation, or *lurp1*, a protein secreted by macrophages that is linked to embryogenesis, through preventing formation of the fused, wedge-shaped trough that is a precursor to heart tube formation (9). Thus, macrophages are positioned to shape the myocardial layer and remain in proximity during remodeling of the developing heart. In the case of rodents, apoptotic debris has been observed in macrophages of the developing rat heart, likely acquired through the physiological processes of vestigial structure deletion, cell number control, and structure remodeling, suggesting that phagocytic signaling could modulate growth cues for early cardiac morphogenesis (10). In mice, the developing heart contains multiple macrophage subsets, which can be classified into distinct populations based on the expression of C-C chemokine receptor 2 (CCR2) and are derived from yolk sac, recombination activating gene 1^+^ lymphomyeloid, and fetal Fms-like tyrosine kinase 3^+^ monocyte lineages (11). Functionally, CCR2^−^ yolk sac-derived macrophages were found to be required for coronary development and maturation, whereas macrophages derived from lymphomyeloid and fetal monocyte lineages appeared dispensable for normal heart development. Mechanistically, embryonic CCR2^−^ macrophages demonstrated increased expression of insulin-like growth factor (IGF) ligands, a proangiogenic signal, compared to CCR2^+^ macrophages, and were selectively recruited to perfused vasculature where they functioned to remodel the developing coronary vascular plexus by promoting expansion of perfused blood vessels. Despite no overt role for lymphomyeloid and fetal monocyte lineages for heart development in this study, the differences in timing of recruitment, location within the developing heart, and transcriptional profiles indicate the need for additional studies to understand whether these distinct macrophage lineages contribute to embryonic and postnatal organ development, or in response to embryonic cardiac developmental insults.

## Nonphlogistic Phagocytic Clearance During the Cardiac Steady State

In adults, efficient cell removal is critical for ensuring that the daily turnover of senescent cells does not disturb the steady state by inciting inflammation. During steady state, both professional phagocytes and non-professional “bystander” cells may participate in removal and metabolism of dead cells through the process of efferocytosis (12). Apoptosis eliminates senescent cells in the absence of inflammation, as efferocytic mechanisms suppress proinflammatory cytokines (13). In the heart, a recent analysis of cell generation and turnover revealed that cardiomyocyte numbers are initially established perinatally and appear to be constant throughout human life; cardiomyocyte turnover was estimated at <1% per year in adulthood (14). This was in contrast to endothelial and mesenchymal cells, including fibroblasts and smooth muscle cells, which exchanged at a high rate. Thus, in comparison to other cell types, cardiomyocyte apoptosis is not a likely significant factor in daily macrophage phagocytic programming. This does not rule out however, that cardiomyocytes, through release of degradation products through lysosomal exocytosis (15), exosomes, or ectosomes, in turn may stimulate receptor mediated endocytosis or phagocyte signaling toward the maintenance of the steady state. For example, ectosomes released by some cell types can induce phagocytic receptor anti-inflammatory pathways in macrophages (16). In the adult murine heart at steady state, resident macrophages are maintained by local proliferation to populate and replicate within the myocardium (4). Resident CCR2^−^ macrophages can be divided into MHCII^HI^ and MHCII^LO^ subsets, which differ significantly in gene ontology of antigen processing pathways (4). In a scenario where mice express the fluorescent TdTom reporter, strictly in cardiomyocytes (Rosa-TdTom x Mlc2V-cre), increased fluorescence can be found associated with resident cardiac macrophages. Although this could be associated with macrophage phagocytosis during preparation of cardiac extracts, it does support the prospect of cardiomyocyte sampling as a means for communication between myocytes and macrophages. *In vitro*, MHCII^LO^ cardiac macrophages were most efficient at taking up dead cell cargo (4, 17). Many interesting questions remain in terms of homeostatic clearance in the heart. For example, it is unclear whether specific receptors are utilized in the steady state, as opposed to during cardiac inflammation or injury, as well as the associated relationships to anti-inflammatory pathways. Furthermore, the classification and characterization of cardiac resident macrophages in these studies and others discussed herein are derived from the murine heart. Whether these observations will translate to the human heart remains to be determined.

## Phagocytic Clearance During Cardiac Infection

A potential yet understudied role for resident cardiac macrophages in the steady state is the defense against infection. As proof of principle, injection of fluorescently labeled bacteria leads to uptake by cardiac phagocytes (18). *Streptococcus pneumoniae* enters the myocardium and forms damaging microlesions (19); however, these lesions exhibit relatively low levels of inflammatory infiltrate that only increase after antimicrobial therapy. In the case of Chagas heart disease, the leading cause of infectious myocarditis and caused by the protozoan parasite *Trypanosoma cruzi*, infection can be characterized by cardiomyocyte necrosis throughout the course of disease (20), likely inducing activation of cardiac macrophages. Another important feature of experimental infection with *T. cruzi* is the massive increase in apoptotic, activation-induced cell death in CD4^+^ T lymphocytes (21). Phagocytosis of these apoptotic lymphocytes by macrophages results in macrophage secretion of TGF-β leading to suppressive TGF-β signaling and increased growth of *T. cruzi* in the macrophage (22, 23). Interestingly, in patients with cardiac clinical forms of Chagas disease, there is an increase in the expression of CCR5 on CD4^+^ T cells, which controls leukocyte migration into the inflamed heart (24), and while CCR5 expression is required during the acute phase for protection against experimental *T. cruzi* infection in mice, it is dispensable for the chronic phase of infection (25). Thus, during the chronic phase of infection, continuous recruitment of CD4^+^ T cells to the infected heart followed by their apoptosis and engulfment by cardiac macrophages could contribute to an immunosuppressive environment to allow *T. cruzi* to escape host responses leading to chronic cardiomyopathies. Similar to Chagas disease, patients with infectious endocartitis due to *Coxiella burnetti*, can exhibit valvuopathy with increased levels of apoptotic leukocytes. This has also been linked to efferocytic anti-inflammatory macrophage polarization, thereby permitting increased bacterial replication (26). In contrast, anti-inflammatory macrophages play an important role in limiting excessive inflammation during viral myocarditis (27). Following coxsackievirus B3 infection, viral myocarditis was milder in female mice, which displayed enhanced expression of anti-inflammatory mediators by macrophages, compared to male mice, which displayed higher levels of proinflammatory macrophage markers. Adoptive transfer of *ex vivo* alternatively activated macrophages alleviated the excessive inflammation in male mice, consistent with macrophage polarization contributing to the extent of myocardial inflammation. These studies highlight that cardiac macrophages likely play an important role in shaping host defense against a variety of pathogens in the heart and this is further supported by the ability of pathogens, such a *T. cruzi*, to exploit essential phagocyte function to evade clearance.

## Phagocytic Clearance as an Inducer of Phagocyte Programming of Cardiac Repair after Acute Ischemic Injury and Clinical Reperfusion

In Western Societies, including the United States, heart disease and stroke remain leading causes of death (28). Patients who survive their first heart attack have an increased risk of secondary MI, heart failure, and stroke, and secondary risk is linked to the local and systemic inflammation that occurs after first MI (29). A key function of recruited and mobilized leukocytes at site of infarction is the degradation and phagocytosis of dying and necrotic cells, and extracellular matrix. Inhibition of innate immune cells is associated with adverse outcomes post-MI (30). Similar to inflammatory atherosclerosis (31, 32), the infarction consists of a necrotic core (33) that can expand between the endocardium and epicardium. Bordering this necrotic core are endangered cardiomyocytes that may be either salvaged or not, dependent in part on the efficiency of the repair process. This is necessary for subsequent fibrogenic responses and remodeling to compensate for lost cardiomyocytes, as well as angiogenesis to reperfuse the tissue. Recent data directly link efferocytosis by inflammatory immune cells (12) to wound healing in the myocardium and implicate phagocytosis receptors on monocytes and macrophages as a key link between inflammation resolution and organ function (34, 35). In the elderly, suboptimal dying-cell clearance may lead to a non-resolving inflammation (36), and maladaptive cardiac repair, thereby accelerating heart failure (37). An additional clinical component is the contribution of reperfusion, which although restores oxygen supply, can also itself facilitate reperfusion-associated injury (38). Below we expand on key steps surrounding phagocytic clearance after cardiac wound injury.

## Chemotaxis Signals for Phagocytes to Sites of Myocardial Injury

Directed chemotaxis to the infarction proceeds by trafficking through a gradient of reducing oxygen tension and a chemotactic gradient of local so-called apoptotic *find-me* signals (39), including lipids, such as lyso-phosphatidyl-choline (LPC) and sphingosine-1-phosphate (S1P). LPC is externalized and excreted during apoptosis (40, 41) and amasses during ischemia in the heart *via* thrombin activation of Ca2^+^-independent phospholipases (42), consistent with its role as a *find-me* signal in the damaged heart. S1P, another lipid *find-me* signal is produced by sphingosine kinase 1 (SPHK1). Apoptotic stress induces SPHK1 activation, which can then promote S1P secretion (43). In addition to lipid *find-me* signals, proteinaceous tissue recruitment factors include fractalkine (CX3CL1), which is cleaved by caspase-3 during apoptosis. Released fractalkine interacts with CX3CR1 on macrophages for cell recruitment (44). Fas/CD95-induced chemokines, which includes monocyte chemoattractant protein 1/C-C chemokine ligand 2 (MCP-1/CCL2), can recruit monocytes and monocyte-derived macrophages for phagocytosis *via* CCR2 (45). Nucleotides ATP and UTP from apoptotic and necrotic cells also likely act as *find-me* signals in the myocardium. In apoptotic cells, the plasma membrane channel pannexin 1 (PANX1) may act as a portal for nucleotide release (46). During ischemia, cellular stress increases glycosylation of PANX1 and increased ATP release from myocytes to activate fibroblast transformation (47). ATP can also serve as a signal for neutrophil chemotaxis *via* purinergic P2Y2 and A3 adenosine receptors *in vitro* and *in vivo* (48). Knockdown of *P2y2* inhibits migration (49), all consistent with ATP released from PANX1 acting as a *find-me* signal in the heart. Taken together, a variety of “find me” signals may be released by apoptotic cells in the heart but whether these signals cooperate or are distinct and how they direct the phagocytic response during cardiac injury require further investigation.

## Unique Functions of Phagocyte Subsets During Cardiac Repair Post MI

Following MI, innate immune cells are recruited and mobilized to heart to clear damaged tissue and initiate cardiac repair. Neutrophils, the recruitment of which is linked to circadian oscillation (50), accumulate in the ischemic myocardium in large numbers within a few hours, acting as first responders after the onset of injury (51). Neutrophils act to clear necrotic debris, but are also capable of clearing apoptotic cells in other circumstances (52). While neutrophils are among the first to arrive in the injured heart, their function has often been associated with detrimental effects on heart healing. For example, blockade of neutrophil function has been shown to limit adverse ventricular remodeling and preserve systolic function (53, 54) and the magnitude of the neutrophil response was predictive of adverse outcomes in both mice (50) and humans (55, 56). Initial studies using antibody-mediated depletion of neutrophils revealed protective effects during myocardial ischemia–reperfusion injury (IRI) (57–59). However, in the context of the prolonged ischemia that occurs after experimental, permanent coronary ligation, neutrophil depletion led to increased cardiac fibrosis and progressively worsened cardiac function with increased markers of heart failure (57). The worsened outcome following neutrophil depletion was attributed to reduced phagocytic receptor *Mertk* gene expression on cardiac macrophages, preventing efficient clearance of dying cardiomyocytes and proper inflammation resolution. Mechanistically, neutrophil gelatinase-associated lipocalin was identified as a neutrophil secreted molecule that was capable of programming macrophages toward a highly phagocytic, MerTK-expressing, proreparative phenotype. In addition to secreted factors, neutrophils represent a large population of short-lived inflammatory cells that undergo apoptosis in the infarcted myocardium. Phagocytosis of apoptotic neutrophils by macrophages directs inflammation resolution by promoting an anti-inflammatory program leading to the release of proresolving mediators such as IL-10, TGF-β, lipoxins, and resolvins (13, 60) and also contributes to the maintenance of homeostasis by imprinting tissue resident macrophages with an anti-inflammatory phenotype in various tissues throughout the body (61). It has been proposed that phagocytosis of apoptotic neutrophils by cardiac macrophages promotes inflammation resolution in the infarcted myocardium (51). Therefore, depletion of neutrophils might be expected to worsen repair by limiting phagocytosis-dependent reprogramming of macrophages toward a reparative phenotype. However, additional studies are required to directly assess this in the heart. Thus, neutrophils likely contribute to repair after myocardial infarction through the secretion of soluble mediators, which promote the differentiation of reparative macrophages, but also by acting as a direct trigger for phagocytosis-dependent, anti-inflammatory pathways in macrophages.

**Figure 1:**
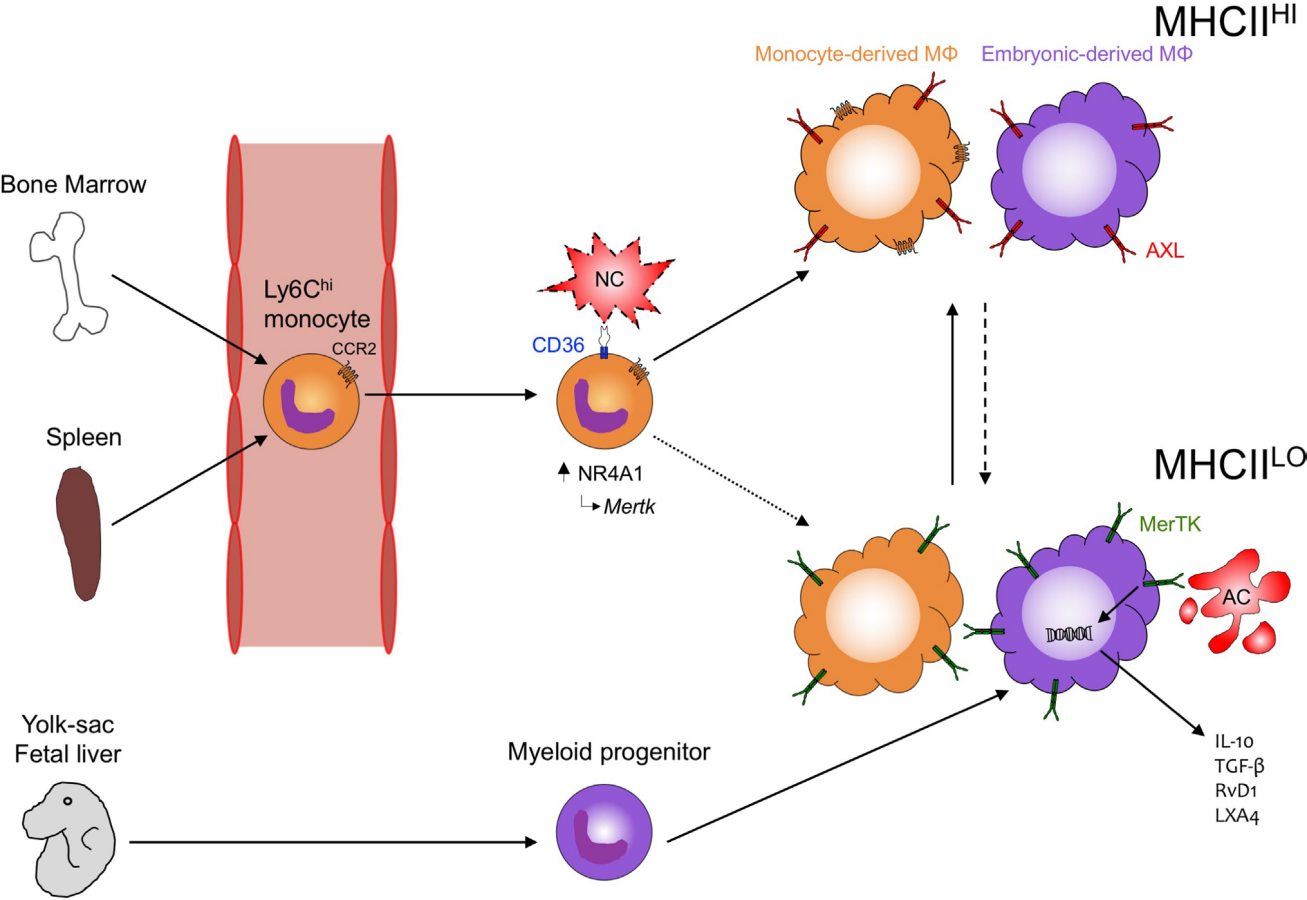
Phagocytosis in the heart. During development, embryonic yolk-sac-derived and then fetal liver-derived macrophages seed the heart with MHCII^LO^ MΦ that can later differentiate into MHCII^HI^ MΦ. Following cardiac injury, both recruited phagocytes (Ly6C^hi^ monocytes) and resident (embryonic-derived MΦ) contribute to tissue repair and inflammation resolution. CCR2 mediates recruitment of Ly6C^hi^ monocytes to the injured myocardium where CD36 expression on Ly6C^hi^ monocytes leads to the recognition and clearance of necrotic cardiomyocyte (NC) debris and the subsequent induction of NR4A1-dependent transcription and reprogramming to MerTK-expressing, monocyte-derived MΦ. Peripheral blood monocytes preferentially differentiate into MHCII^HI^CCR2^+^ MΦ in the heart but whether these cells also become MHCH^LO^CCR2^−^ MΦ (dotted line) requires further investigation. Concurrently, MHCIl^LO^ embryonic-derived MΦ recognize apoptotic cardiomyocytes (ACs) through MerTK leading to the production of anti-inflammatory cytokines (IL-10, TGF-β) and specialized, proresolving lipid mediators (RvD1, LXA4). MHCII^HI^ embryonic-derived MΦ may also recognize ACs through MerTK and other phagocytic receptors, such as AXL and differentiate into proresolving MHCH^LO^ MΦ (dashed line). How monocyte-derived MΦ and embryonic-derived MΦ interact and the mechanisms regulating differentiation between the different MHCII-expressing populations remain understudied and will likely have important consequences toward recovery after cardiac injury.

After neutrophil numbers peak in the infarcted mouse myocardium, Ly-6C^hi^ monocytes (Figure 1 Working Model) accumulate in response to CCL2 and exhibit proteolytic and phagocytic functions to degrade and clear the damaged myocardium (62). Ly-6C^hi^ monocytes engulf dying cardiomyocytes (63) and in other contexts, are able to efferocytose and cross-present cell-associated antigens (64). In the heart, Ly6C^hi^ monocytes give rise to proliferative Ly6C^low^ macrophages, and this requires the nuclear receptor protein NR4A1 (65). Interestingly, NR4A1 is also linked to *Mertk* gene expression (63) and therefore as an expected consequence, NR4A1 deficiency in macrophages has been shown to impair engulfment and clearance of apoptotic cells (66). Macrophage polarization may also be important in cardiac wound healing, as alternatively activated macrophages have been linked to enhanced efferocytosis (67) and repair of the infarcted adult murine heart (68). There are multiple inducers of anti-inflammatory macrophage polarization, including cytokines IL-4 and IL-13 (69), which transduce their effects through IL-4 and IL-13 receptors, including the common IL-4Rα subunit (70). Administration of IL-4 increases survival and improves cardiac function after MI, however, in *Trib1-*deficient mice, which exhibit impaired alternative macrophage polarization, these mice are not protected by IL-4 (68). Complete deficiency of IL-13 in male mice decreases survival and impairs cardiac remodeling after myocardial infarction (71). Additionally, the combination of IL-4 or IL-13, together with apoptotic cells, promotes macrophage tissue repair (72).

While these observations have advanced our understanding on how apoptotic cell engulfment reprograms macrophage function and how this in turn informs phagocyte function during cardiac injury, many of these studies require moving beyond the generalized M1/M2 macrophage polarization paradigm to comprehend deeper relationships between macrophage polarization and function (73). This is further emphasized by the identification of a variety of macrophage subsets residing in the myocardium of differing developmental origins, which changes over the course of development and aging or following cardiac injury (4, 74, 75). Of the cardiac resident macrophage populations examined to date, all appear capable of phagocytosing cardiomyocytes (4, 17), highlighting the potential of these different phagocytes to participate in the wound healing process. However, many questions remain on whether the different populations have distinct or overlapping function and whether their function differs under varied pathophysiological conditions, such as sterile would healing or host defense. Emerging evidence indicates that embryonic-derived cardiac macrophages may be superior at mediating inflammation resolution and tissue repair following cardiac injury (74). The progressive loss of these cells with age may also explain in part the adverse outcomes that occur in adult humans during cardiovascular disease (75), and lead to the identification of novel therapeutic avenues to reverse the clock and recapture the protective responses of embryonic-derived macrophages. For example, embryonic-derived macrophages rely on macrophage colony-stimulating factor (M-CSF) signaling through CSFR1 as both a survival and self-renewal signal (76), and injection of M-CSF, but not granulocyte colony-stimulating factor (G-CSF), increases collagen content to accelerate infarct repair and attenuate left ventricular dysfunction (77), suggesting that M-CSF-mediated preservation of embryonic-derived macrophages may improve repair after cardiac injury. At the time of this publication, significantly more studies are warranted on examining the different macrophage subsets in the heart both at steady-state and during the many forms of cardiac disease.

## Phagocytosis by Non-Professional Phagocytes

While phagocytosis of microbes or apoptotic cells in the heart is predominantly promoted by macrophages, other non-professional phagocytes have been shown to participate in this process. Interestingly, cardiomyocytes themselves can phagocytose latex particles *in vitro* (78) and potentially cardiomyocyte debris *in vivo* (79, 80), the latter of which may have an important role in the developing heart. Recently, myofibroblasts were identified as another non-professional phagocyte that were capable of engulfing apoptotic cardiomyocytes (81). Myofibroblast-mediated clearance of dying cells after myocardial infarction was dependent on milk fat globule epidermal growth factor (MFG-E8), which was produced in part by myofibroblasts, and mice lacking MFG-E8 displayed increased inflammation and adverse tissue remodeling. Furthermore, macrophages, through the release of microvesicles, can alter the type of particles engulfed by non-professional phagocytes, to in turn affect the inflammatory response. For example, efferocytes release IGF-1, which upon recognition by non-professional phagocytes such as epithelial cells, reduces the size of particulate uptake (82). While the presence of professional cardiac phagocytes, such as macrophages, minimalizes the necessity for non-professional phagocytes, such as myofibroblasts, to phagocytose a dying neighbor, the contribution of non-professional phagocytes to the clearance of apoptotic and necrotic debris is underexplored and whether these cells cooperate with macrophages in the heart to promote cardiac repair requires additional study.

## Phagocytic Receptor Targets and Stimuli in Heart

There are multiple targets in addition to dying cardiomyocytes that can activate phagocytic receptor signaling in the heart, including clearance of red blood cells, a process known as erythrophagocytosis. Intramyocardial hemorrhage is a frequent complication in ST-Elevation Myocardial Infarction (STEMI) patients reperfused by primary percutaneous coronary intervention. STEMI patients with intramyocardial hemorrhage also frequently present with residual myocardial iron that is associated with adverse left ventricular remodeling and suggestive of ongoing inflammation (83). In canines, myocardial iron deposits were directly related to proinflammatory burden with iron deposits found directly in cardiac macrophages (84). The accumulation of iron in macrophages is likely a direct consequence of excessive erythrophagocytosis in the hemorrhage. Iron overloading of macrophages has been shown to induce a proinflammatory activation state characterized by TNF-α and toxic hydroxyl radicals release, which can then lead to premature senescence of resident fibroblasts and impaired wound healing (85). Similar to intramyocardial hemorrhage, erythrocyte-rich thrombi also contain more inflammatory cells leading to impaired reperfusion in STEMI patients (86). In addition to erythrophagocytosis, the thrombus includes platelets and fibrin that are processed by phagocytes and for which the mechanisms of removal remain unclear. Macrophages are capable of phagocytosing platelets leading to the induction of iNOS (87), which may contribute to matrix degradation and adverse ventricular remodeling. A recent report also identified macrophages as important mediators of fibrin clearance with CCR2^+^ macrophages constituting the majority of cells engulfing fibrin (88). This has important implications for the heart, where CCR2^+^ macrophages are present early in cardiac development and expand in numbers after injury.

In the case of necroptosis, a regulated, nonapoptotic form of necrotic cell death, signaling through receptor-interacting protein kinase-3 and mixed lineage kinase-like proteins leads to externalization of phosphatidylserine, a prophagocytic “*eat me*” signal (89), and an opportunity for phagocytes to recognize and clear these “necrotic bodies” to limit inflammation in the injured heart (90). Given the relatively long-lived life cycle of adult differentiated cardiomyocytes, it is logical to speculate that antiphagocytic, or so-called “*don’t-eat-me*” signals (39), may be important in warding off macrophage-mediated elimination. Indeed, *don’t-eat-me* signals, which include CD31 and plasminogen activator inhibitor I, prevent viable cells from engulfment by phagocytes (91). The most widely studied *don’t-eat-me* signal is CD47, which is a membrane protein expressed on the surface of most cells and has been shown to prevent tumor cells from immunologic removal (92). CD47 interacts with SIRPα on phagocytes, recruits phosphatases, and inhibits downstream activation of the phagocyte actin cytoskeleton, thereby preventing engulfment (93, 94), and has been associated with blocking recognition of prophagocytic molecules, such as calreticulin (93). It has been shown that CD47 is expressed in abundance on apoptotic neonatal cardiocytes (95), and mice lacking thrombospondin-2, a CD47 ligand, exhibit impaired cardiomyocyte survival and dilated cardiomyopathy leading to higher mortality (96). However, whether the aforementioned requires CD47, or whether CD47 is directly involved in removal of apoptotic cells in the heart, is unknown. Recently, CD47-blocking antibodies have been effective at restoring defective atherosclerotic phagocytosis (97, 98) and preventing atherosclerosis in experimental mouse models (99). More recent studies (100), suggest that early targeting of CD47 in the myocardium after infarction may be a new viable strategy, in combination with current standards of care, to enhance the efficacy of wound repair in the ischemic heart, and specifically through promotion of enhanced cardiomyocyte phagocytosis. However, the titration of anti-CD47 antibodies will likely need to be optimized to prevent phagoptosis of live cells (101).

The clearance of apoptotic cells and cellular debris is also mediated by soluble mediators of the acute-phase response including pentraxins and complement. The long pentraxin, PTX3, has been observed in the myocardium and increases in the blood of both humans (102) and mice (103) after MI. PTX3 has been shown to bind to apoptotic cells limiting activation of the first component of the classical complement pathway, C1q, and inhibiting their phagocytosis by dendritic cells (104). In contrast to dendritic cells, PTX3 increased macrophage phagocytosis of apoptotic cells (105), indicating that PTX3 may redirect apoptotic cell phagocytosis during injury to promote inflammation resolution and limit self-antigen presentation. The cumulative effect for the actions of PTX3 are cardioprotective as PTX3-deficient mice display exacerbated heart damage with increased cardiomyocyte apoptosis and complement activation after MI (103), and administration of exogenous PTX3 ameliorated cardiomyocyte apoptosis and inflammation in a heart transplantation model (106). Circulating levels of the classical short pentraxin, C-reactive protein (CRP), are also elevated in the blood of humans after MI (102). Like PTX3, CRP is also able to promote apoptotic cell clearance by binding to oxidized phosphorylcholine on the apoptotic cell surface leading to recognition and phagocytosis by macrophages (107, 108). While both elevated and insufficient levels of CRP have been linked with disease progression in a variety of autoimmune disorders (109), the increased levels of CRP observed after MI in humans is believed to promote complement activation in the infarct leading to increased cardiomyocyte death (110). Consistent with a detrimental role for CRP after cardiac injury, selective apheresis of CRP reduced infarct size in pigs after MI (111), and administration of human CRP, which binds to damaged cells and activates complement, enhanced infarct size in rats after MI (112). Inhibition of complement activation in rabbits reduced infarct size after cardiac IRI (113), suggesting that regulation of complement activation by PTX3 and CRP may control the extent of damage after cardiac injury.

Finally, the biodegradation of collagen by phagocytes and the deposition of new extracellular matrix is formative during the final stages of tissue remodeling. Macrophages are capable of phagocytosing collagen with M2-like macrophages predominating collagen uptake *in vivo* in a mannose receptor (CD206)-dependent pathway (114). Whether collagen phagocytosis stimulates macrophages to promote extracellular matrix deposition remains unclear; however, loss of CD206^+^ M2-like macrophages during MI and the resultant catastrophic decrease in collagen deposition (68), underscores the importance of macrophages shaping the extracellular matrix during the final stages of tissue remodeling. Importantly, phagocytes can fine-tune their response according to the size and source of the phagocytic target. A recent finding indicated that reactive oxygen species localization may be one signal that regulates this response with smaller microbes triggering ROS intracellularly in neutrophils and larger microbes triggering extracellular release of ROS, effectively adapting the immune response to the microbe size (115). This may be particularly relevant in the heart where macrophages encounter apoptotic targets of varying size during wound healing ranging from the diminutive red blood cell to the relatively larger cardiomyocyte, which is many fold larger in surface area relative to the macrophage.

## Recognition of the Cardiac Parenchyma by Phagocyte Receptors

The recognition of “eat-me” signals on apoptotic cells is performed by a variety of conserved recognition receptors, which either directly or indirectly recognize the apoptotic cell and often display redundancy in the “eat-me” signals recognized. In the heart, early reports have linked apoptotic cell recognition by scavenger receptors (SRs) in cardiac repair. For example, mice deficient in class A scavenger receptor (SR-A) exhibit increased myocardial rupture after infarction resulting in part from excessive inflammation (116). Whether SR-A deficiency impairs phagocytosis of dying cardiomyocytes by macrophages is unclear; however, the hearts of SR-A-deficient mice display evidence of increased cardiomyocyte necrosis (117), which could be the consequence of secondary necrosis following impaired apoptotic cell clearance. Interestingly, SR-A deficiency reduced myocardial IRI and this was associated with increased microRNA-125b expression and reduced apoptosis in macrophages (118). In contrast to permanent occlusion MI, reperfusion spares resident cardiac macrophages that would otherwise be subject to ischemic-induced cell death (119), so the attenuated injury in SR-A-deficient mice after IRI may be due to the actions of preserved resident macrophage function. CD36, another SR, also appears important for wound healing after myocardial injury (120), particularly early after the onset of injury (63). Within hours after MI, uptake of apoptotic and necrotic cardiomyocyte debris was mediated by CD36 on Ly6C^hi^ monocytes and the importance of CD36-mediated clearance by Ly6C^hi^ monocytes was revealed in CD36-deficient bone marrow recipients, which displayed increased infarct size early after MI compared to WT recipients (63). CD36-mediated engulfment was also found to induce the expression of NR4A1, which is required to mediate the differentiation of Ly6C^hi^ monocytes into reparative Ly6C^lo^ macrophages (65). The protective effects mediated by CD36 may be limited by its proteolytic degradation as CD36 levels decreased after MI in WT but not in matrix metalloproteinase (MMP)9-deficient mice (120). Preservation of CD36 in MMP-9-deficient mice increased macrophage phagocytosis of apoptotic neutrophils, improving inflammation resolution and LV function. Efferocytosis of apoptotic cardiomyocytes has been shown to require MerTK to resolve acute inflammation and permit cardiac repair after permanent occlusion (34, 35) and clinically relevant myocardial reperfusion (17). Additionally, combined deficiency of MerTK and MFG-E8 in macrophages impaired efferocytosis-linked vascular endothelial growth factor (VEGF)-A secretion, worsening angiogenesis and cardiac repair after MI (34). MerTK and additional receptor tyrosine kinase family members, Tyro3 and AXL, indirectly recognize apoptotic cells through bridging molecules growth-arrest-specific 6 and protein S, which bind phosphatidylserine. Galectin-3 has been suggested as a new, putative MerTK ligand (121), and consistent with this role, Galectin-3-deficient mice had increased infarct size and worsened ventricular function after MI (122). While the role of MerTK in cardiac repair is well characterized, roles for either Tyro3 or AXL in the heart are currently unknown. Overall, the phagocyte is equipped with a variety of receptors capable of recognizing apoptotic cells. How these receptors mediate engulfment, the signals that regulate their expression in the heart during both homeostasis and disease, and in many cases, the ligands these receptors recognize on the surface of apoptotic cells remain unknown and are the focus of current investigations.

## Cardiac Consequences of Phagocytosis-Dependent Intracellular Signaling and Reprogramming

The engulfment of foreign bodies by phagocytes triggers signal transduction cascades beyond the necessary cytoskeletal and phago-lyosomal processing pathways that are required to physically internalize and digest extracellular-derived material. In the case of microbial phagocytosis, phagosomes have been shown to recruit pH-lowering caspase-1, which was activated by the NLRP3 inflammasome and ROS signaling, leading to cross-presentation of phagocytosed bacterial antigens (123). In the case of macrophages that have ingested apoptotic cells, intracellular signaling culminates in inhibition of proinflammatory cytokine production and secretion of anti-inflammatory mediators (13). Such signaling pathways remain an active area of investigation. One key family of efferocytosis-signaling molecules are the nuclear receptors. For example, the nuclear receptor, liver X receptor (LXR), is activated upon apoptotic cell engulfment and can in turn promote further efferocytic events through the induction of efferocytic receptors (124). *In vitro*, loss of LXR reduced macrophage-mediated efferocytosis and subsequently impaired the tolerogenic effects that result from apoptotic cell engulfment, and *in vivo*, LXR-deficient mice exhibited a break in self-tolerance, developing autoantibodies and autoimmune glomerulonephritis. Some of this reprogramming may occur through so-called apoptotic cell response elements (ACREs) (125). With respect to IL-10, apoptotic cell engulfment induces binding of the transcription factor, pre-B cell leukemia transcription factor (Pbx)-1, to the IL-10 promoter and deletion of the Pbx-1 promoter binding site reduces promoter activity and IL-10 production. Interestingly, Pbx-1 deficiency did not completely ablate apoptotic cell-induced IL-10 production, indicating the likelihood of additional transcription factors or ACREs regulating apoptotic cell-induced IL-10 expression. Additional signals from the local milieu also translates into both phenotypic and functional properties of phagocytes. For example, tissue-resident macrophages display unique enhancer landscapes beyond what may be explained by developmental origin and this is determined in part by the tissue microenvironment (3). Transfer of mature, peritoneal macrophages into the lung resulted in upregulation of lung macrophage-specific genes and downregulation of peritoneal macrophage-specific genes in the transferred macrophages, indicating macrophages can be reprogramed by the tissue microenvironment. Phagocytosis itself imprints phagocyte heterogeneity in a tissue-specific context, and though tissue residence defines core macrophage signatures, the function of phagocytosis overlays an additional anti-inflammatory profile (61). Relative to other tissues, such as the lung, cardiac-specific imprinting after phagocytosis has not been fully explored.

## Metabolic Processing of Cleared Myocardial Tissue by Immune Cells, and Links to Cardiac Repair

Tissue injury generates heightened levels of apoptotic and necrotic debris and matrix remnants that once cleared by phagocytes, must be metabolized. Despite the current interest in immunometabolism, the relevance of this process in the heart by immune cells is largely unexplored. For example, emerging roles for metabolism have been linked to stem cell development (126), cell proliferation (127), and T-cell activation (128). In particular, mitochondrial metabolism has been linked to many key macrophage functions, including inflammasome activation (129), bacterial defense (130), and polarization (131). Given that macrophages can engulf cardiomyocytes and associated debris and cardiomyocytes may have both denser cellular and elevated mitochondria content (132), it is reasonable to suspect that following engulfment, macrophages need to increase cellular metabolism to process this large metabolic load and that this in turn influences phagocyte intracellular signaling and reprogramming.

In contrast to traditional viewpoints that metabolic reprogramming occurs solely in response to nutrient or oxygen availability, newer studies reveal that intracellular metabolism is further linked to receptors of damage-associated molecular patterns (DAMPs), which are present in abundance after myocardial infarction (133). In response to LPS, macrophages increase glycolysis and the pentose phosphate pathway, and reduce oxidative phosphorylation despite the presence of abundant molecular oxygen (131, 134, 135). Approaches utilizing glucose tracers demonstrate conservation of this glycolytic shift in response to other proinflammatory stimuli such as IFN-γ and DAMPs (136). Mechanistically, integrated transcriptional and metabolic network analyzes revealed that proinflammatory macrophages have a so-called “broken TCA cycle,” where the truncation of isocitrate dehydrogenase and succinate dehydrogenase (SDH) leads to an accumulation of succinate (137). The increase in succinate stabilizes hypoxia inducible factor (HIF)-1α resulting in an increase in reverse electron transport and ROS production from complex I of the electron transport chain and favoring glycolysis by promoting phosphofructokinase isoform conversion (135, 138). Metabolomic studies also revealed that itaconate modulates proinflammatory macrophage metabolism and effector function by inhibiting the oxidation of succinate to fumarate by SDH (139). Furthermore, HIF-1α may also directly be stabilized by ROS generated during IRI driving a metabolic shift in macrophages toward glycolysis and the subsequent proinflammatory polarization (140).

While DAMPs and hypoxia may polarize cardiac macrophages toward a glycolysis-dominated, proinflammatory profile early during myocardial injury, increased oxygen tensions due to angiogenesis and increased levels of lipids from engulfed apoptotic debris may promote a metabolic shift toward fatty acid oxidation (FAO) through the mitochondria. In this context, alternatively activated macrophages induced by IL-4 consumed more oxygen (141) and this increase in oxidative metabolism was required for the anti-inflammatory phenotype, as inhibition of FAO with the carnitine palmitoyltransferase (CPT)-1 inhibitor, etomoxir, inhibited IL-4 induced alternative macrophage polarization (131). However, another group contrasted CTP-2 requirements by showing that CPT-2-deficient macrophages can still fully polarize toward an alternatively activated macrophage phenotype after IL-4 stimulation, despite inhibition of FAO. Thus, the effect of etomoxir on macrophage polarization might be partially due to off target effects (142). Additionally, few processes are all or none and another recent study reported glucose requirements during alternative macrophage polarization, which was dependent on a mTORC2/Stat6/IRF4 signaling axis (143). Still the evidence to date largely supports a role for mitochondrial oxidative phosphorylation in anti-inflammatory responses as IL-10 can alter macrophage function by promoting mitophagy of damaged mitochondria to support oxidative phosphorylation and limiting glucose uptake and glyocylosis to oppose inflammatory metabolic reprogramming (144). As IL-10 is actively produced in macrophages after efferocytosis, it is worth exploring whether efferocytosis influences cellular metabolism to promote IL-10 production or whether macrophage secretion of IL-10 after efferocytosis functions in an autocrine manner to affect macrophage metabolism. Metabolism of small molecules such as amino acids and vitamins are also involved in macrophage activation. For example, l-arginine-derived metabolites are important mediators for inhibiting the production of TNF-α in mouse splenic macrophages after intestinal obstruction (145). Vitamin A has also been shown to be required for the phenotypic conversion of IL-4 activated macrophages within tissue resident macrophages of the peritoneal cavity (146). Besides its contribution to alternative macrophage activities, lipid metabolism also likely contributes to macrophage phagocytosis by fulfilling its energetic needs and regulating the membrane fluidity that is required for phagocytosis (147). Other links to mitochondrial pathways include mitochondrial UCP2, which is required for continuous uptake of apoptotic cells (148). Taken together, many of the metabolic links between phagocytosis and macrophage function remain unknown, especially in the heart, and discoveries made in the field of immunometabolism as it pertains to the macrophage will likely influence our understanding of inflammation resolution after cardiac injury and inform new therapeutic strategies.

## Cardiac Lymphatics in Immune Surveillance and Tissue Homeostasis

**Figure 2:**
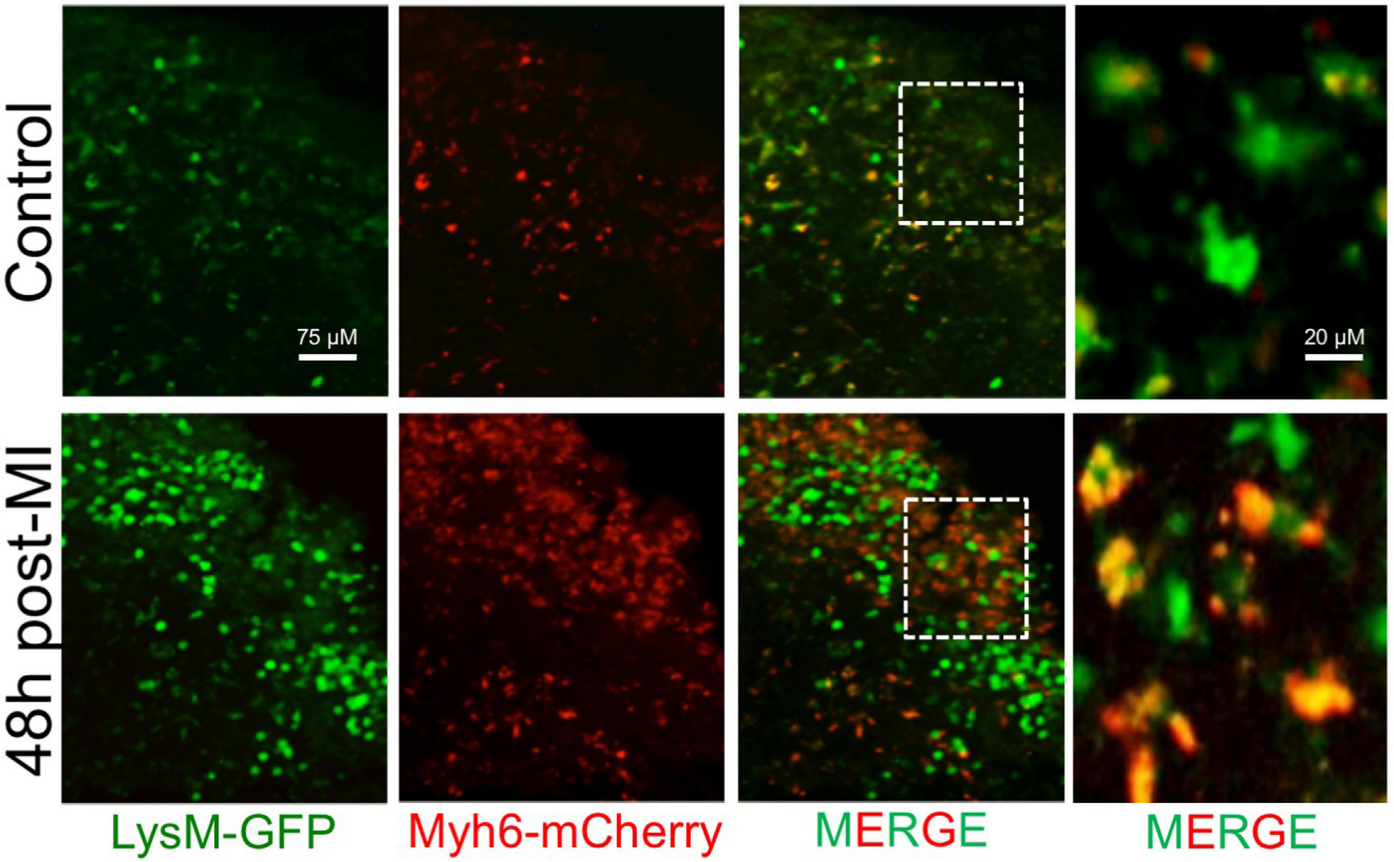
Accumulation of phagocyte-associated cardiac antigen in the mediastinal lymph nodes (MLN) after MI. Myeloid cells (LysM-GFP^+^) traffic cardiac antigen derived from α-myosin heavy chain in cardiomyocytes (Myh6-mCherry^+^) to the MLN during steady-state in mice. Following MI, there is an increase in the number of phagocytes and the amount of phagocyte engulfed cardiac antigen in the MLN.

Recent evidence has demonstrated crucial roles for the lymphatic vasculature of the heart in both immune surveillance and tissue-fluid homeostasis. Under steady-state conditions, the lymphatic network provides a path for dendritic cells to constitutively phagocytose apoptotic cell remnants and transport this self-antigen to T-cell areas in draining lymph nodes, contributing to peripheral self-tolerance (149). In the heart, IRF8-dependent conventional dendritic cells phagocytose the cardiac self-antigen, α-myosin, and transport it to, and present it in, the heart-draining mediastinal lymph node (MLN) where it promotes the induction of α-myosin-specific CD4^+^ T regulatory cells to maintain tolerance (150). Indeed, and in our own hands, trafficking of cardiac antigen to lymph nodes appears to be found in phagocytes (Figure 2 Lymphatics). During inflammation, an elevated number of phagocytes can traffic from the site of injury and carry phagocytosed antigen to the draining lymph nodes (151). Although the cessation of the phagocyte response in inflamed infarct tissue may occur primarily through local cell death, some phagocytes traffic from the infarct tissue to lymphatic organs (119). With respect to DCs, MI results in massive maturation and expansion of all DC subsets in the heart, including monocyte-derived DCs, followed by trafficking and presentation of cardiac-derived antigens to CD4^+^ T cells in the MLN (150). Macrophages also utilize lymphatic vessels to traffic antigen and modulate inflammatory responses in draining lymph nodes and distal sites (152). For example, macrophages take part in reverse cholesterol transport through lymphatics with ablation of these pathways leading to heightened atherosclerosis and inflammatory disease (153). In the heart, direct labeling of cardiac resident macrophages, but not Ly6C^hi^ monocytes, by intramyocardial injection of a cell tracking dye demonstrated that cardiac macrophages constitutively traffic to the MLN, spleen, and bone marrow under steady-state conditions (18). Following MI, the percentage of labeled macrophages doubles in both the spleen and the bone marrow; however, the significance of this migration remains unknown.

With respect to tissue-fluid homeostasis, recent research has demonstrated that VEGF-C-dependent lymphatics expand in the border zone post-MI with further induction of lymphangiogenesis, through VEGF-C injection, leading to increased measures of cardiac function and repair (154, 155). VEGF-C-induced lymphangiogenesis led not only to improved myocardial fluid balance through resolution of tissue edema but also to attenuated cardiac inflammation, in part through egress of DCs and macrophages from the wounded heart. Interestingly, VEGF-C secreting macrophages are implicated in several pathologies and inflammatory processes. In anti-inflammatory tumor environments, macrophages are able to secrete VEGF-C and increase lymphatics, causing downstream tumor metastasis (156). As demonstrated in murine models of lung damage, intestinal bowel disease, and in corneal inflammation, macrophages are able to secrete VEGF-C to induce lymphangiogenesis, regulate lymphatics, and migrate through lymphatics into lymph nodes (157, 158). In the heart specifically, macrophages secrete VEGF-C in murine models of hypertension and when activated by tonicity enhanced binding protein, an osmotic stress responsive transcription factor, contribute to the adaptive response in maintaining interstitial fluid and blood pressure homeostasis (159). Furthermore, macrophages were shown to not only modulate lymphangiogenesis, but also directly interact and remodel lymphatic vessel structure and function. In such cases, macrophages closely interact with lymphatics, possibly incorporating into the vessels, and ultimately augmenting the branching of newly forming lymphatics (160). While efferocytosis has been linked to macrophage production of VEGF-A after MI (34), it is currently unknown whether efferocytosis plays a role in VEGF-C production by macrophages.

## Phagocytosis Links to Cardiac-Specific T-Cell Responses

Professional phagocytes, such as macrophages and dendritic cells, play a critical role in bridging innate and adaptive immunity, which is important in the context of host defense (discussed above). However, after sterile inflammation, such as myocardial infarction, anticardiac T- and B-cell responses can develop suggesting that phagocytosis by cardiac macrophages and DCs can initiate autoimmune responses. Myocardial infarction induces activation and proliferation of CD4^+^ T cells in a cardiac antigen-specific manner, as mice with CD4^+^ T cells specific to an irrelevant antigen fail to mount a response (161). This process is exacerbated in the presence of additional pathophysiology, such as type I diabetes. In both mice susceptible to type I diabetes and type I diabetic patients, myocardial infarction induces postinfarction autoimmunity specific for cardiac antigens such as α-myosin heavy chain, α-actinin-2, and troponin I (162). This is due in part to a lack of central tolerance to cardiac antigens (163), and impaired efferocytosis in individuals susceptible to type I diabetes. High glucose conditions impair macrophage-mediated efferocytosis (164), likely contributing to liberation of self-antigens in an inflammatory context. More recently, infarct lysate-primed, tolerogenic dendritic cells improved remodeling and cardiac function after MI by affecting regulatory T-cell and macrophage polarization (165). Interestingly, tolerogenic dendritic cells migrated only to the regional lymph node near the site of injection but were still able to induce a systemic activation of MI-specific regulatory T cells. In contrast to tolerogenic DCs, conventional and myeloid-derived DCs infiltrate and mature in the infarcted heart and migrate to the MLN, where it was demonstrated that IRF4-dependent conventional DCs were superior in presenting α-myosin to CD4^+^ T cells (150). Despite the importance of IRF4- and IRF8-dependent conventional DCs in presenting cardiac antigens in the MLN, loss of either subset did not impair α-myosin-specific CD4^+^ T-cell responses. Perhaps this is due to the massive presence of monocyte-derived DCs in the MLN after MI. While monocyte-derived DCs were shown to be inferior in generating α-myosin-specific CD4^+^ T-cell responses at either steady-state or after MI, it has been previously reported that this can be overcome by MHC class I/peptide transfer to bystander DCs (166). Importantly, transient autoimmune reactions to cardiac myosin after MI appear to be relatively common among the general population (167), necessitating a better understanding of how phagocytes drive postinfarction autoimmune responses.

## Phagocyte-Linked Myocyte Regeneration

Although not a feature of the adult mammalian heart, the ability to regenerate damaged tissue is common to many multicellular organisms and tissues. For example in the skin and liver, apoptotic cells, prior to engulfment, release growth signals to stimulate the proliferation of progenitor cells (168), and during skeletal muscle injury, cooperation between skeletal phagocytes and satellite cells leads to myocyte regeneration (169). The latter results in part through recognition of phosphatidylserine on apoptotic myoblasts by brain-specific angiogenesis inhibitor 1 on healthy myoblasts promoting fusion between healthy myoblasts to form myotubes. Similar to the heart, macrophages have also been demonstrated to participate in the tissue repair process of damaged skeletal muscle with macrophage depletion by clodronate-containing liposomes leading to prolonged clearance of necrotic myofibers and impaired skeletal muscle regeneration (170). Some of the mechanisms can be attributed to generalized tissue repair processes, where macrophages acquire an anti-inflammatory phenotype characterized by gene expression of IL-10, IL-13 receptor, arginase 1 (in mice), and other factors. In skeletal muscle, macrophage polarized gene expression requires CREB, as mice with conditionally mutated promoters exhibit severe defects in muscle fiber regeneration (171). Additionally, both heart and skeletal muscle injury leads to recruitment of Ly6C^hi^ monocytes that ultimately give rise to anti-inflammatory Ly6C^lo^ macrophages (65, 172). However, tissue-specific differences likely exist that shape the role for phagocytes in repair after injury as CCR2 deletion or antagonism reduces adverse ventricular remodeling and improves ventricular function after MI (173, 174), but impairs myogenesis following skeletal muscle injury (172, 175). For example, recruited phagocytes are a critical source of IGF-1 that is needed to promote muscle regeneration following skeletal muscle injury (175), but in the heart, embryonic-derived resident macrophages may be the critical source for this growth factor (11).

Despite some similarities in the tissue repair process, the adult heart possesses poor regenerative potential in contrast to the regenerative capacity of skeletal muscle and relative to reports in the neonatal mouse heart, where injury can stimulate cardiomyocyte proliferation (176). In the adult heart, immune-mechanisms of tissue replacement largely leads to fibrosis and therefore loss of full cardiac contractile potential. In a genetic model of cardiomyocyte cell death, neonatal mice expanded a population of embryonic-derived resident cardiac macrophages, which generated marginal inflammation and promoted cardiac recovery after cardiomyocyte proliferation and angiogenesis (74). Similarly, macrophages were required for neonatal heart regeneration and neoangiogenesis after MI with macrophages from P1 hearts promoting angiogenesis essential for cardiac regeneration compared to macrophages from P14 hearts, which produced factors repairing the damaged tissue but also stimulating fibrotic scar formation (177). Thus, understanding the context specific molecular cues that empower regenerative potential versus scarring is of critical clinical importance in the heart. Cardiospheres and cardiosphere-derived exosomes have shown promise for cardiac regeneration and some of these pathways may signal through apoptotic cell receptors (178, 179). Recent clinical trials suggest that yet more work is to be done in the field of cardiosphere-derived therapy to translate findings from mouse to man (180).

## Phagocyte Function During Heart Failure and Associated Systemic Factors

Nonresolving inflammation is a driver of disease and a hallmark of many cardiovascular syndromes including heart failure (36). In both animal models of heart failure and in humans with end-stage heart failure, there is evidence of ongoing cardiomyocyte apoptosis indicating that continued clearance of dying cardiomyocytes by phagocytes and the subsequent reprogramming of these efferocytes may influence the progression of this disease (181–183). During chronic heart failure, macrophages continue to increase in numbers due to increased local macrophage proliferation and differentiation of recruited monocytes into macrophages with each population displaying distinct gene expression patterns (73). Limiting the expansion of monocyte-derived macrophages through blockade of monocyte recruitment preserves ejection fraction after MI (73), indicating that altered phagocyte function contributes to heart failure. Similarly, in a model of pressure overload-induced heart failure, ICAM1-deficient mice have decreased monocyte recruitment and exhibit no overt signs of cardiac fibrosis and minimal ventricular dysfunction (184). ICAM1 has been linked to suppression of efferocytosis with ICAM1 deficiency in macrophages promoting efferocytosis of apoptotic cells (185). Increased efferocytosis by ICAM1-deficient macrophages led to increased expression of IL-10, which has been shown to attenuate pressure overload-induced hypertrophic remodeling (186), indicating that enhanced efferocytosis by cardiac resident macrophages may contribute to the protective response to heart failure in ICAM1-deficient mice. Galectin-3 represents another marker of altered phagocyte phenotype during heart failure as it is expressed only by myocardial macrophages in failure-prone hypertrophied hearts but not normal hearts, where it has been shown to contribute to cardiac dysfunction in rats (187) and be predictive of adverse events in human heart failure patients (188). In mice lacking Galectin-3, myocardial fibrosis and macrophage infiltration were reduced with preservation of left ventricular function during chronic angiotensin II-induced hypertension demonstrating a cardiac-deleterious role for Galectin-3 (189). Galectin-3 plays a critical role in phagocytosis by macrophages (190), but it can also be proteolytically cleaved by MMP to release a soluble protein (191), which is capable of inducing fibroblast proliferation and collagen production (187). Whether Galectin-3-dependent phagocytosis or production of soluble Galectin-3 by macrophages contributes to the progression of heart failure remains to be determined; however, Galectin-3 influences macrophage polarization *in vitro* (192), suggesting that Galectin-3 alters cardiac macrophage function in the failing heart. Additional phagocytic receptors, such as SR-A, have been implicated in regulating phagocyte function during heart failure with SR-A-deficient macrophages displaying increased expression of proinflammatory genes following LPS-stimulation *in vitro* and adverse vascular remodeling during angiotensin II-induced hypertension *in vivo* (193).

The link between phagocyte function and heart failure is likely a consequence of both local pathological changes within the myocardium itself and pathophysiologies in distant organ systems that feedback on the heart and also manifest as systemic changes. For example, cardiorenal syndrome manifests as impaired renal function following MI characterized by increased infiltration of macrophages into the kidney and elevated renal levels of TGF-β and T-cell immunoglobulin and mucin domain (TIM)-1 associated with the onset of renal fibrosis (194). TIM-1 has been linked to efferocytosis (195), and overexpression of TIM-1 in mice leads to the development of spontaneous and progressive interstitial kidney inflammation with fibrosis (196), demonstrating that enhanced expression of apoptotic receptors distal to the site of injury may have deleterious effects. In the context of hypertension, a recent report has demonstrated an interplay between neurohormonal modulation of phagocyte function before the onset of hypertension, leading to excessive inflammation by the phagocyte system and contributing to the development of hypertension (197). Heart failure also has systemic consequences and in a recent report, mice subjected to myocardial pressure overload in turn activated a heart–brain–kidney network that required phagocyte function in both the heart and kidney and culminated in activation of cardiac-resident Ly6C^lo^ macrophages to mediate the adaptive response (198). Following pressure overload in the heart, sympathetic nerve activation led to activation of renal collecting-duct cells, which through interactions with renal macrophages led to the release of CSF2 into the circulation by endothelial cells within the kidney. Within the overloaded heart, CSF2 expanded and activated Ly6C^lo^ macrophages to secrete amphiregulin inducing a cardiac hypertrophic response. Interestingly, a parabiosis model revealed that these cardiac-resident Ly6C^lo^ macrophages increased in number largely through *in situ* proliferation; however, further examination of the Ly6C^lo^ resident macrophage population using well-established markers MHCII and CCR2 or lineage tracing was not performed. This is of particular importance as embryonic-derived resident cardiac macrophages have been shown to decline with age (75) and embryonic-derived resident cardiac macrophages may be superior at mediating adaptive responses in the heart (74). How the different macrophage subsets change during the course of disease and whether phagocytosis of ongoing cardiomyocyte death alter the progression of heart disease and its related sequela remain to be determined.

## Phagocytosis Post Heart Transplantation and New Therapeutic Opportunities for Tolerance

Transplant rejection involves both innate and adaptive immune responses. Clinical progress has reduced acute cardiac transplant rejection, however, beyond ten years, complications of immunologic intervention often lead to significant comorbidities, particularly posttransplant vasculopathy. Continuous immune-suppression during transplant raises risks of opportunistic infections, and of hematologic (199), metabolic, and nephrotoxic side effects (200). Interestingly, acute phagocytosis and innate inflammation during allograft IRI has been linked to chronic pathophysiology. For example, perioperative and acute inflammation are prognostic for worse long-term transplant outcome (201, 202). Graft reperfusion may trigger reperfusion-associated cell death (38) and cell necrosis occurs during allograft cold storage and continues in allograft reperfusion. Both of these processes liberates allo-antigens in an inflammatory context. Efficient clearance of dead cells by macrophages prevents these self-antigens from becoming immunogenic debris and can actively initiate tissue-reparative and tolerogenic signaling (203) and as a consequence, natural defects in phagocytosis have been correlated with, but not yet causally linked, to poor outcomes posttransplant (204). Similar to post-MI, cardiac allograft rejection and tolerance are regulated by phagocyte subsets. After IRI, cardiac graft rejection is linked to elevated Ly6C^hi^ monocytes (205, 206) with both alloantigen-dependent and -independent factors contributing to immune cell activation (207). Ly6C^hi^ monocytes differentiate into Ly6C^lo^ macrophages and antigen presenting cells, which recognize allogenic non-self and contribute to graft injury (208, 209) through cytokines and T-cell activation (210, 211). However, not all macrophage function is detrimental, as some macrophage subsets belong to the heterogeneous classification of myeloid-derived suppressor cells (MDSCs), which can accumulate in allografts, suppress effector T cells, and induce tolerance (212–214). For example, anti-CD40L mAbs (clone MR1) promote experimental cardiac tolerance through suppressive DC-SIGN^+^ macrophages (215). Separately, a unique strategy harnesses *natural* immune-regulatory properties of efferocytosis: apoptotic donor *sp*lenocytes, fixed with the chemical cross-linker 1-*e*thyl-3-(3′-dimethylaminopropyl)-*c*arbo*d*i*i*mide (ECDI-SPs) (216), are engulfed by macrophages to induce transplant tolerance (217). In the heart, transfusion of ECDI-SPs from the donor strain prior to heart transplantation dramatically prolongs survival of the heart graft and tolerance induction is dependent on phagocytosis of the apoptotic cells (218) and signaling through apoptotic cell receptors. This process enhances the accumulation of MDSCs in both the spleen and the cardiac allograft, which limit the activation and recruitment of antiallograft CD8^+^ T cells (219), and also appears to involve alterations of antigen presenting cell costimulatory ligands (220). Alloantigen-presenting plasmacytoid dendritic cells have also been shown to mediate tolerance to vascularized grafts (221). The complete molecular mechanisms by which uptake of apoptotic splenocytes induce cardiac allograft survival remain unclear.

## Molecular Modulators and Inhibitors of Cardiac Phagocytosis

Accumulating molecular evidence suggests that the clearance efficiency of the cardiac infarct is not optimal and therefore amenable to potential therapeutic intervention. For example, cardiomyocytes induce macrophage receptor shedding to suppress phagocytosis (222). The mechanism involves the activity of ADAM proteases, which recognize the efferocytosis receptor, MerTK and execute its proteolytic shedding from the surface of macrophages (223). ADAM17-mediated proteolytic degradation releases a soluble protein (solMER), which is believed to compete with membrane-bound MerTK in the binding of bridging molecules on the surface of apoptotic cells antagonizing MerTK-mediated efferocytosis. Additionally, loss of MerTK from the cell surface also eliminates MerTK-induced anti-inflammatory responses. For example, MerTK signaling increases the ratio of cytoplasmic-to-nuclear 5-lipoxygenase promoting the production of specialized proresolving mediators (SPMs), including LXA_4_ and RvD1 (60). In the heart, production of SPMs initiates a proresolving response actively promoting inflammation resolution with administration of RvD1 during MI reducing neutrophil recruitment to the spleen and infarct and promoting anti-inflammatory polarization of macrophages culminating in reduced fibrosis and preserved ventricular function (224). Efferocytosis and SPM production is impaired under conditions where MerTK is cleaved and introduction of a cleavage-resistant MerTK in mice improves inflammation resolution during both peritonitis (60) and myocardial reperfusion (17). Previous studies have also shown that the SR CD36 is susceptible to cleavage during atherosclerosis (225) and by MMP-9 after myocardial infarction (120) and our own work recapitulates these findings (63). Of course proteolysis is not specific to macrophages as TLR7/8 activation in neutrophils reduces immune complex phagocytosis through shedding of FcgRIIA (226). On the target cell side, molecules of the CD47/SIRP1α axis have also been associated with proteolysis susceptibility. In vascular smooth muscle cells, CD47 was shown to be a target of MMP-2-mediated proteolytic degradation (227) and in mice, MMP-2 deficiency led to enhanced survival after MI (228). While CD47 cleavage was not examined in the MMP-2-deficient mice, a greater number of cardiomyocytes persisted in the infarct and border zone.

Other common comorbidities such as diabetes mellitus and hyperlipidemia in patients with heart failure have also been linked to both apoptotic receptor shedding and impaired phagocytosis. Macrophage exposure to diabetic conditions *in vitro*, leads to reduced miR-126 expression and a concomitant increase in its direct target, ADAM9 (164). Similar to ADAM17, ADAM9 is also able to mediate the cleavage destruction of MerTK and impair efferocytosis of apoptotic cardiomyocytes. Human heart tissue from diabetic patients with heart failure recapitulated the *in vitro* findings with human diabetic failing heart tissue exhibiting reduced miR126 and increased ADAM9 expression with a reduction in phosphorylated MerTK, a surrogate marker for MerTK signaling, indicating that antagonism of MerTK-mediated processes under diabetic conditions could translate to an increase in adverse clinical outcomes in heart failure patients. With respect to hyperlipidemia, apolipoprotein E (apoE)-deficient mice, which develop hyperlipidemia and atheroscleorosis when maintained on a high-fat diet, exhibit impaired wound healing after MI (62). The dysregulated healing response may be due in part to effects of hyperlipidemia on apoptotic cell clearance (229). In vascular smooth muscle cells, oxidized low-density lipoprotein *in vitro* or hyperlipidemia *in vivo* impaired phagocytosis of apoptotic cells leading to the development of secondary necrotic cells capable of releasing both IL-1α and IL-1β further propagating the inflammatory response. Mice maintained on a high-fat diet also displayed increased activation of ADAM17 on the vascular endothelium, indicating that proteolytic degradation of apoptotic receptors may contribute to impaired phagocytosis during obesity and hyperlipidemia (230). Targeting hyperlipidemia using atorvastatin, restored phagocytic function to retinal pigment epithelial cells treated with cholesterol crystals or oxidized low-density lipoproteins (231), demonstrating a proof-of-principle approach to mitigating detrimental effects of hyperlipidemia on phagocytosis in the heart.

Hypoxia is another common environmental stress after coronary artery occlusion. The activation of proteases such as ADAM17 have been linked to hypoxia and HIFs (232), indicating that the hypoxic myocardium may also antagonize phagocytosis by promoting ADAM17-mediated proteolytic degradation of apoptotic receptors. However, in the absence of detectable receptor shedding, hypoxia has also been shown to suppress efferocytosis by macrophages and this was due in part to reduced gene expression of efferocytic receptors during hypoxia exposure (233). While phagocytes encounter varying oxygen tensions during development, migration, and infiltration of tissues, hypoxia was shown to be dispensable for macrophage differentiation, at least in the hypoxic tumor microenvironment (234), suggesting that oxygen tension may act to fine-tune macrophage function. HIFα subunits are the critical regulators of phagocyte function during hypoxia and inflammation, controlling metabolism, cytokine production, migration, and survival and thus, likely shape the phagocyte response during wound healing in the hypoxic heart. In the infarcted myocardium, macrophages have been shown to express both HIF-1α and HIF-2α (235); however, our understanding of the timing and functional significance of HIFα subunits in phagocytes during cardiac injury is incomplete. Knockdown of HIF-1α in the hematopoietic compartment improved LV function after MI and this was attributed to reduced recruitment of neutrophils and monocytes to the infarcted myocardium (236). In contrast to the more widely studied HIF-1α isoform, less is known about HIF-2α function in macrophages. A recent study implicated a role for HIF-2α in redox control and phagocytosis by macrophages during normoxic conditions (237). Elevations in mitochondrial ROS in HIF-2α-deficient macrophages led to nuclear translocation of NRF2 and NRF2-dependent transcriptional induction of the phagocytic receptor, MARCO, which translated into increased macrophage phagocytic function. Similar to HIF-1α, HIF-2α has also been implicated in regulating macrophage LPS-induced secretion of proinflammatory cytokines and chemokines (238), though this occurred independent of changes in cellular energy homeostasis, in contrast to the connection of HIF-1α to glycolysis. Given the importance of both HIF-1α and HIF-2α in secretion of proinflammatory mediators by macrophages, it is tempting to speculate that myeloid cell expression of HIFα subunits may play a detrimental role in the hypoxic heart. However, HIFα isoforms show differential activation in macrophages with HIF-1α induction following M1 polarization and HIF-2α induction following M2 polarization (239), the latter of which leads to macrophage expression of arginase 1 and attenuated inflammation during obesity-induced insulin resistance in adipose tissue (240). HIFα subunits also play an important role in the adaptive angiogenic and lymphangiogenic responses to hypoxia, both of which have been implicated in heart healing after MI. Here again, HIF-1α and HIF-2α may have opposing roles in phagocytes with HIF-1α promoting and HIF-2α antagonizing the angiogenic effects of VEGF (241). Additional studies are needed to fill the large gaps that remain in our understanding on how oxygen tension and HIFα subunits regulate phagocytosis in the heart. Taken together, a number of risk factors promote inefficient clearance of dying cells during cardiovascular disease, leading to secondary necrosis and prolonged inflammation. Future studies identifying the mechanisms of how these risk factors conspire to antagonize phagocytosis in the heart will lead to the identification of new targets for the development of novel therapeutics to promote wound healing in the heart.

## Future Prospects for the Therapeutic Targeting of Phagocytic Repair and Regenerative Pathways in the Heart

Improvements in clinical treatment have led to reduced mortality after first MI. Nevertheless, the incidence of heart failure, including after MI, is on the rise. Current clinical trials, including the CANTOS trial, have demonstrated the merits of anti-inflammatory therapy for the reduction of secondary events (242, 243). Given the critical role of efferocytosis and phagocytosis after MI, this pathway represents a tractable target amenable to modulation during the time patients are in hospital and during the formative stages of disease progression. Approaches that strategize to enhance phagocytic efficiency are appealing due to the multiple checks and balances that are naturally built in to mechanisms of phagocytosis and therefore minimize off target effects; that is engulfment requires both the downregulation of “don’t eat me” signals, as well the converse presentation of prophagocytic “eat me” ligands. One approach is to use cardiosphere-derived cells or exosomes, which are heart cell products with antifibrotic, anti-inflammatory, and angiogenic properties. Uptake of CDCs by macrophages induces a proresolving phenotype leading to reductions in left ventricular fibrosis and inflammation with improved left ventricular function (178, 179, 244). Alternative strategies have employed liposomes carrying phosphatidylserine, an “eat me” signal that directs efferocytosis, to increase anti-inflammatory cytokine production by macrophages after myocardial infarction (245). Other macrophage-targeted lipid based drug carriers are able to reprogram macrophages to a proresolving phenotype and improve tissue repair and limit infarct expansion (246). With respect to the proteolytic degradation of efferocytic receptors, such as MerTK and CD36 (17, 225), strategies which block the cleavage degradation including cleavage-blocking peptides, may be a viable approach for enhancing protection after cardiac insult. Additionally, since the soluble forms of these efferocytic receptors are increased in the serum after MI, monitoring their levels in humans may serve as a useful biomarker for novel therapeutic interventions. Taken together, targeting efferocytosis and phagocytosis pathways in the heart represents a promising therapeutic strategy to limit inflammation and promote reparative functions in a variety of cardiovascular disease settings.

## Author Contributions

MD, SZ, and ET wrote the manuscript. MD, LG, and ET designed and prepared the figures. KG, IH, EV, KH, and XL provided their expertise in critically reviewing and revising the manuscript. All authors edited the manuscript and approved the final version for publication.

## Conflict of Interest Statement

The authors declare that the research was conducted in the absence of any commercial or financial relationships that could be construed as a potential conflict of interest.
